# Beneficial effects of oral and topical sodium bicarbonate during a battery of team sport-specific exercise tests in recreationally trained male athletes

**DOI:** 10.1080/15502783.2023.2216678

**Published:** 2023-05-25

**Authors:** William H. Gurton, Jordanne Greally, Karolina Chudzikiewicz, Lewis A. Gough, Anthony Lynn, Mayur K. Ranchordas

**Affiliations:** a Sheffield Hallam University, Sport & Physical Activity Research Centre, Health Research Institute, Sheffield, UK; b Birmingham City University, Human Performance and Health Research Group, Centre for Life & Sport Sciences, Birmingham, UK; c Technology & Engineering Sheffield Hallam University, Food Group, College of Business, Sheffield, UK; d Advanced Wellbeing Research Centre, Sheffield, UK

**Keywords:** Supplements, alkalosis, team sports, Yo-Yo intermittent recovery test level 2, repeated sprint ability

## Abstract

**Objective:**

This study examined the effects of oral and topical (PR Lotion; Momentous) sodium bicarbonate (NaHCO_3_) during a battery of team sport-specific exercise tests.

**Method:**

In a block randomized, crossover, double-blind, placebo-controlled design, 14 recreationally trained male team sport athletes performed a familiarization visit and three experimental trials receiving: (i) 0.3 g·kg^−1^ body mass (BM) NaHCO_3_ in capsules + placebo lotion (SB-ORAL), (ii) placebo capsules +0.9036 g·kg^−1^ BM PR Lotion (SB-LOTION), or (iii) placebo capsules + placebo lotion (PLA). Supplements were given ~120 min prior to the team sport-specific exercise tests: countermovement jumps (CMJ), 8 × 25 m repeated sprints and Yo-Yo Intermittent Recovery Level 2 (Yo-Yo IR2). Blood acid–base balance (pH, bicarbonate) and electrolytes (sodium, potassium) were measured throughout. Rating of perceived exertion (RPE) was recorded after each sprint and post-Yo-Yo IR2.

**Results:**

Distance covered during the Yo-Yo IR2 was 21% greater for SB-ORAL compared with PLA (+94 m; *p* = 0.009, *d* = 0.64) whereas performance was only 7% greater for SB-LOTION compared with PLA (480 ± 122 vs. 449 ± 110 m; *p* = 0.084). Total completion time for the 8 × 25 m repeated sprint test was 1.9% faster for SB-ORAL compared with PLA (−0.61 s; *p* = 0.020, *d* = 0.38) and 2.0% faster for SB-LOTION compared with PLA (−0.64 s; *p* = 0.036, *d* = 0.34). CMJ performance was similar between treatments (*p* > 0.05). Blood acid–base balance and electrolytes were significantly improved for SB-ORAL compared with PLA, but no differences were observed for SB-LOTION. Compared to PLA, RPE was lower for SB-LOTION after the fifth (*p* = 0.036), sixth (*p* = 0.012), and eighth (*p* = 0.040) sprints and for SB-ORAL after the sixth (*p* = 0.039) sprint.

**Conclusions:**

Oral NaHCO_3_ improved 8 × 25 m repeated sprint (~2%) and Yo-Yo IR2 performance (21%). Similar improvements in repeated sprint times were observed for topical NaHCO_3_ (~2%), but no significant benefits were reported for Yo-Yo IR2 distance or blood acid–base balance compared to PLA. These findings suggest that PR Lotion might not be an effective delivery system for transporting NaHCO_3_ molecules across the skin and into systematic circulation, therefore further research is needed to elucidate the physiological mechanisms responsible for the ergogenic effects of PR Lotion.

## 1. Introduction

Athletes competing in field-based team sports repeatedly perform high-intensity efforts (i.e. sprints, jumps) interspersed with short rest periods [1]. Repeated sprint ability (RSA) is an important determinant of success in team sports but declines throughout competitive matches [2]. Substantial anaerobic energy demand from completing maximal sprint efforts leads to the accumulation of hydrogen ions (H^+^) within muscles [3], which may cause an intramuscular acidosis. While the deleterious effects of declining pH during exercise are debated [4], a cellular acidosis is suggested to inhibit energy production via anaerobic glycolysis and limit action potentials required for muscle contractions [3,5,6]. These biochemical changes contribute toward skeletal muscle fatigue [7,8], therefore nutritional strategies that restore acid–base balance could prove beneficial during team sport exercise.

Sodium bicarbonate (NaHCO_3_) is an extracellular buffering aid that has been extensively researched [9]. Ingestion of 0.3 g·kg^−1^ body mass (BM) NaHCO_3_ in fluid or capsules 90–120 min pre-exercise raises the concentration of blood bicarbonate (HCO_3_^−^) by ~5.0–6.0 mmol·L^−1^, which elevates the pH gradient between intracellular and extracellular compartments, subsequently upregulating the lactate-H^+^ co-transporter to remove H^+^ from muscles [10]. Additionally, ingesting NaHCO_3_ increases the amount of sodium (Na^+^) in the blood [11,12], which may lead to improvements in hydration status [13]. Considering that NaHCO_3_ ingestion may attenuate losses of intramuscular potassium (K^+^) during exercise [12], these changes in electrolytes could also result in upregulation of Na^+^/K^+^-ATPase activity to limit muscle depolarization and sustain excitation–contraction coupling [8].

An established method for evaluating the ergogenic effect of NaHCO_3_ on team sport-specific exercise performance is the Yo-Yo Intermittent Recovery Test Level 2 (Yo-Yo IR2) [14]. NaHCO_3_ ingestion has improved distance covered during the Yo-Yo IR2 [15,16]. Similar results have been reported in team sport athletes for the effect of NaHCO_3_ on RSA (3 sets, 6 × 20 m) [17]. Interestingly, others have shown no improvements for RSA in rugby [18] and soccer [19] players after 0.3 g·kg^−1^ BM NaHCO_3_. It is possible that gastrointestinal (GI) discomfort commonly associated with NaHCO_3_ ingestion prevented these participants from improving their performance [18,20]. Orally ingested NaHCO_3_ dissociates into Na^+^ and HCO_3_^−^ upon reaching the stomach, where the HCO_3_^−^ neutralizes gastric acid, which generates excessive carbon dioxide production that can induce belching and vomiting [21]. These large amounts of exogenous Na^+^ may also aggravate intestinal mucosa and prompt osmotic fluctuations that can cause diarrhea [18]. Many athletes are deterred from using NaHCO_3_ due to these GI side effects, the poor palatability of fluid beverages and/or the high number of capsules (~35 for a 90 kg athlete) required to achieve an ergogenic dose [22]. NaHCO_3_ supplementation strategies that bypass the GI tract could therefore provide a favorable alternative to oral ingestion [23].

One novel approach is PR Lotion (Momentous, Park City, Utah), which is a topically applied muscle cream that is purported to transport NaHCO_3_ molecules across the skin via a transdermal drug delivery system [24]. PR Lotion adopts an innovative formulation that encapsulates NaHCO_3_ molecules within fatty acid salts that fluidize the outermost layer of skin and modulate tight junctions in the epidermis, allowing NaHCO_3_ molecules to be absorbed into the bloodstream [25]. McKay et al. [26] reported that PR Lotion did not significantly improve average power during 3 × 30 s Wingate cycling tests compared to a placebo (*p* = 0.108). However, they also found no differences in time-to-exhaustion (TTE) cycling performance between oral NaHCO_3_ and PR Lotion (363 ± 80 vs. 349 ± 119 s, *p* = 0.697). Given their lack of a placebo group during the TTE cycling task, it is difficult to conclude whether there was an ergogenic effect of PR Lotion. Furthermore, it is not yet known whether PR Lotion alters the concentration of electrolytes and hydration status to a similar degree as oral NaHCO_3_ ingestion. Additional research comparing oral and topical NaHCO_3_ is needed before conclusions can be drawn regarding the efficacy of PR Lotion. Therefore, the aim of this study was to examine the effects of oral and topical NaHCO_3_ (PR Lotion) during a battery of team sport-specific exercise tests. Our hypothesis was that oral and topical NaHCO_3_ would improve team sport-specific exercise performance (countermovement jumps [CMJ], 8 × 25 m repeated sprints, Yo-Yo IR2) compared with a placebo.

## 2. Materials and methods

### 2.1. Study design

A block randomized, double-blind, placebo-controlled, crossover design was used for this study. Participants were randomly allocated to receive each of the three nutritional supplements (oral NaHCO_3_, topical NaHCO_3_ and placebo) in a counterbalanced order using an online sequence generator (www.randomization.com).

### 2.2. Participants

Our sample size calculation conducted on G*Power (version 3.1.9.4) revealed that 15 participants were needed to achieve statistical power (*β* = 0.80; *α=*0.05). This assumed that repeated measures analysis of variances (ANOVA; within-factors) would be used to analyze performance outcomes, with an expected medium effect size (η_p_^2^ = 0.06). Correlation between repeated measures was estimated from reliability data for our performance outcomes [27]. To account for 10–20% drop out rates, 18 participants were recruited; however, three withdrew due to injury and one because of time constraints. Therefore, 14 recreationally trained male team sport athletes (body mass: 81.9 ± 10.1 kg; stature: 182.0 ± 5.4 cm; age: 26.5 ± 5.8 years, sporting background: 3 × hockey, 7 × soccer, 2 × basketball, 1 × rugby, 1 × cricket) completed the study. Participants received a £25 gift voucher and free supplements after taking part. Eligibility criteria stated that participants performed ≥2 training sessions per week of their sport. They were excluded if they had: (i) previously used NaHCO_3_, (ii) an intolerance to cornflour, and (iii) a medical condition that impacts high-intensity exercise. Ethical approval was obtained from the Institutional Ethics Committee (ER42014516). Participants completed a health questionnaire and provided written informed consent before commencing the study.

### 2.3. Procedures

Participants were instructed to avoid strenuous exercise and alcohol for 24 h prior to sessions. They attended the indoor sports hall on four separate occasions. The first visit was a familiarization session to habituate participants to the team sport-specific exercise tests. During the remaining three sessions, participants completed exercise tests after receiving either oral NaHCO_3_, topical NaHCO_3_ or a placebo. These sessions were separated by 5–7 days to ensure appropriate recovery and washout of treatments. Testing was conducted at the same time of day to control for the confounding effect of circadian rhythms on exercise performance [28]. Participants were asked to wear the same footwear for each session and to replicate their diet 24 h prior to each visit.

### 2.4. Team sport-specific exercise tests

Participants completed a 10-min warm up of jogging, 3 × 10 m sprints, agility drills, stretches, and 3 × 20 m runs at 50%, 70%, and 90% of perceived maximum. After 5 min, they performed a first CMJ using an optimal measurement system (Optojump Next, Microgate, USA). Participants completed one practice and three maximal jumps separated by 1 min. After 3 min, they performed an 8 × 25 m repeated sprint test. Timing gates (Brower timing systems, Draper, Utah, USA) were placed at 0 and 25 m to record sprint times. Following each sprint, participants jogged back to the start line, with sprints departing every 25 s until the protocol had been repeated eight times. Participants received 2 min of recovery and then conducted a second CMJ test. After a further 5 min, they performed the Yo-Yo IR2, which required them to repeatedly carry out 2 × 20 m shuttle runs at increasing speeds dictated by audio signals [14]. Each stage was separated by 10 s recovery where participants jogged around a cone positioned 5 m behind the start line. Test termination was classified as two failures to reach the finish line before the signal, at which point distance covered during the Yo-Yo IR2 was recorded. After 5 min recovery, participants performed a final CMJ test.

### 2.5. Supplementation protocol

During experimental trials, participants performed the team sport-specific exercise tests after receiving: (i) NaHCO_3_ in capsules + placebo lotion (SB-ORAL), (ii) placebo capsules + PR Lotion (SB-LOTION), and (iii) placebo capsules + placebo lotion (PLA). Supplements were prepared by a laboratory technician not involved with the study. Oral NaHCO_3_ was given as a 0.3 g·kg^−1^ BM dose in size 0 vegetarian capsules (Your Supplements, Stockport, UK). An equal number of capsules (31 ± 4) containing cornflour was used as a placebo for SB-LOTION and PLA. Cornflour is an inert substance that effectively blinds NaHCO_3_ [22]. Capsules were filled using a capsule filling device (ALL-IN Capsule, USA) and contained either ~0.8 g NaHCO_3_ (Health Leads Ltd, UK) or ~0.4 g cornflour (Sainsbury’s, UK). These were checked for weight and administered as three equal doses with 7 mL·kg^−1^ BM water at 15-min intervals across a 30-min period [29] commencing 135 min before the team sport-specific exercise tests. Participants applied PR Lotion to their legs and lower back as a 0.9036 g·kg^−1^ BM dose during the final 15 min of this 30-min supplementation period. PR Lotion was administered 120 min prior to the team sport-specific exercise tests as evidence suggests peak changes in muscle/blood pH occur ~120 min after applying PR Lotion [30]. PR Lotion is ~33% NaHCO_3_; therefore, a 0.9036 g·kg^−1^ BM dose was chosen to theoretically match the amount of NaHCO_3_ given during SB-ORAL and SB-LOTION. The placebo lotion was matched for all ingredients except NaHCO_3_ and both lotions were provided in plastic tubs. Supplements were given alongside a carbohydrate-rich meal (1.5 g·kg^−1^ BM; biscuits, wholegrain cereal bars, cornflakes with milk) to standardize food consumed prior to testing and minimize GI discomfort after NaHCO_3_ ingestion [29].

### 2.6. Experimental trials

Baseline urine samples were analyzed for color using an 8-point Likert scale [31] and osmolality using an osmometer (Vitech Scientific, Partridge Green, UK). Capillary blood samples (95 μL) were analyzed for acid–base balance (pH, HCO_3_), electrolytes (Na^+^, K^+^), hemoglobin, and hematocrit using a blood gas analyzer (i-STAT Alinity, Abbott, USA). Plasma volume was estimated from hemoglobin and hematocrit concentration [32]. Additional 20 μL blood samples were analyzed for lactate using a Biosen C-Line (EKF Diagnostics, Cardiff, UK). Visual analog scales (VAS) for eight GI side effects were completed to quantify aggregate GI discomfort [11,33].

Supplements were administered across a 30-min window. Participants then completed VAS and treatment assignment questionnaires that asked them to select which treatment they believed had been given (“oral NaHCO_3_,” “topical NaHCO_3_,” “placebo,” “unsure”) and explain their reasons [22]. Participants rested for a further 85 min, before blood and urine samples were taken pre-warm-up. They also repeated VAS and treatment assignment questionnaires and rated on 1–5 Likert type scales how much they expected the supplement they thought had been given to improve performance (“1” = no expectations, “5” = extremely high expectations).

**Figure 1.**
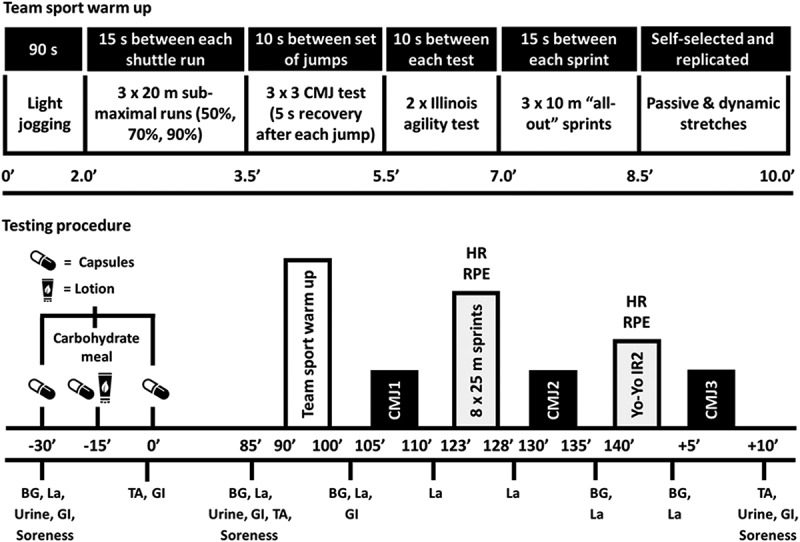
Experimental schematic showing timings (minutes) and procedures for warm up and battery of team sport-specific exercise tests. BG = blood gas (pH, bicarbonate, hemoglobin, hematocrit, sodium, potassium), La = blood lactate, GI = gastrointestinal discomfort questionnaire, TA = treatment assignment questionnaire.

After the 10-min warm-up, blood samples were repeated. Participants commenced the team sport-specific exercise tests 120 min after applying PR Lotion (~105 min after the final set of capsules). Additional blood samples were analyzed for lactate after CMJ tests and the repeated sprints. Rating of perceived exertion (RPE; 6–20 Borg scale) was recorded after each sprint and post-Yo-Yo IR2. Blood sampling was repeated pre- and post-Yo-Yo IR2. Post-exercise urine samples, treatment assignment questionnaires, and VAS were completed after the final CMJ test. An overview of experimental procedures is shown in Figure 1.

### 2.7. Statistical analysis

Statistical analyses were performed using SPSS 26.0 for Windows (IBM, Chicago, IL) and Exploratory Software for Confidence Intervals (ESCI, https://thenewstatistics.com/itns/esci/). Grouped data and standardized residuals were assessed for normality using Shapiro–Wilks tests. Homogeneity of variance/sphericity were analyzed using Mauchly tests and any violations were corrected via Greenhouse–Geisser adjustments. Two-way repeated measures ANOVA were used to determine treatment * time interactions for sprint completion times, CMJ performance, blood metabolites, urine osmolality and RPE during the repeated sprint test. All other normally distributed outcome measures were assessed using one-way repeated measures ANOVA. When significant effects were found, post hoc pairwise comparisons were made using Bonferroni correction factors. Effect sizes were calculated using SPSS for *η*_*p*_^*2*^ and ESCI for Cohen’s *d*. These were interpreted using the classifications of 0.01, 0.06, and 0.14 as small, medium, and large effect sizes for *η*_*p*_^*2*^ and 0.2, 0.5, and 0.8 as small, moderate, and large effect sizes for Cohen’s *d* [34]. Friedman tests were used to assess treatment effects for non-normally distributed data (GI discomfort, urine color, expectations), with Chi-square (χ^2^) reported as the test statistic. When significant effects were found, post hoc pairwise comparisons were conducted, with median and *Z* values presented. Treatment assignment ratings (“correct,” “incorrect”) were analyzed using 2 × 2 χ^2^ tests to determine blinding efficacy. Mean differences and *95% CI* are reported for treatment comparisons. Data are presented as mean ± SD (unless stated) and statistical significance was set at *p* ≤0.05.

## 3. Results

### 3.1. Team sport-specific exercise performance

**Figure 2.**
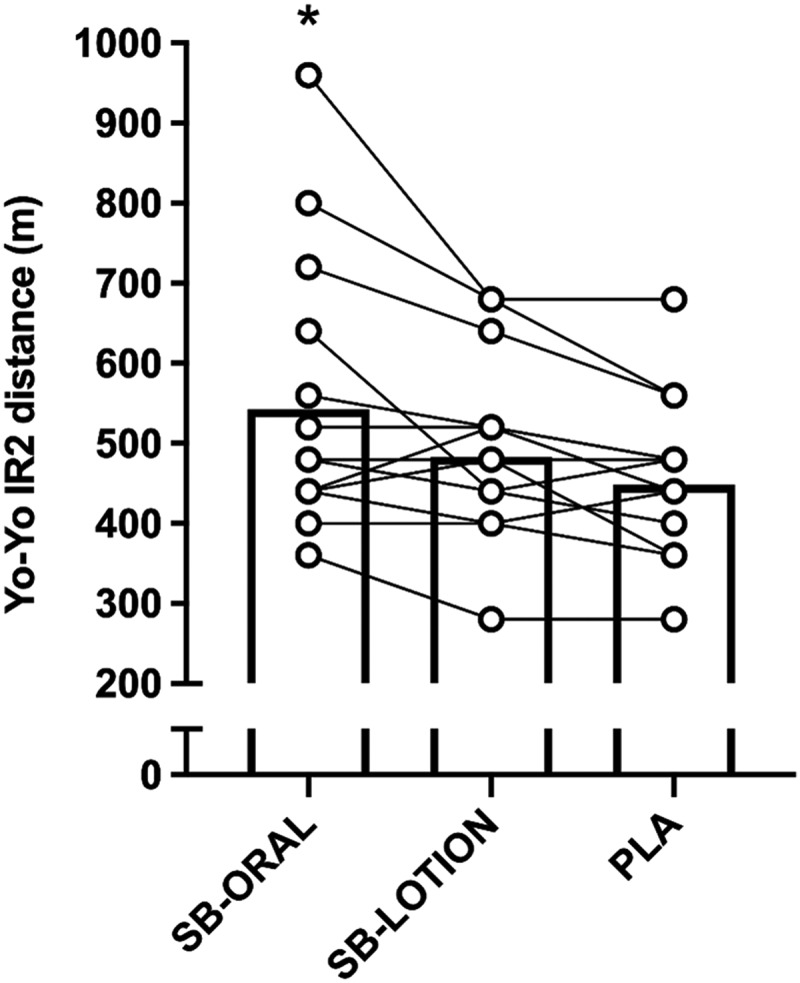
Total distance covered during the Yo-Yo IR2 test. Bars represent mean values. Individual treatment differences depicted by symbol/line. SB-ORAL = oral sodium bicarbonate, SB-LOTION = topical sodium bicarbonate, PLA = placebo; * greater than PLA (*p* < 0.05). .

There was an effect of treatment on Yo-Yo IR2 performance (*p* = 0.001, *η*_*p*_^*2*^ = 0.414; Figure 2). Total distance covered was 21% greater for SB-ORAL compared with PLA (+94 m; *95% CI*: 23, 166; *p* = 0.009). Cohen’s *d* revealed a medium effect of SB-ORAL (*d* = 0.64; *95% CI*: 0.21, 1.05). Although not statistically significant, the total distance covered was 13% greater for SB-ORAL compared with SB-LOTION (+63 m; *95% CI*: −8, 134; *p* = 0.089; *d* = 0.41) and 7% greater for SB-LOTION compared with PLA (+31 m; *95% CI*: −3, 66; *p* = 0.084; *d* = 0.27).

There was no significant treatment * time interaction for individual sprint times during the repeated sprint test (*p* = 0.490, *η*_*p*_^*2*^ = 0.069) but there was an effect of treatment (*p* = 0.024, *η*_*p*_^*2*^ = 0.250). Significant differences in completion times for individual sprints are shown in Table 1. Cohen’s *d* revealed medium effects of SB-ORAL during the fifth (*d* = 0.59, *95% CI*: 0.19, 0.97), sixth (*d* = 0.52, *95% CI*: 0.14, 0.87), and eighth (*d* = 0.61, *95% CI*: 0.10, 1.10) sprints. Cohen’s *d* revealed a medium effect of SB-LOTION during the eighth (*d* = 0.56, *95% CI*: 0.14, 0.97) and small effects during the fifth (*d* = 0.47, *95% CI*: 0.09, 0.83) and sixth (*d* = 0.49, *95% CI*: 0.14, 0.83) sprints. Completion times for individual sprints were similar for SB-ORAL and SB-LOTION (all *p* > 0.05; Table 1).

**Table 1.** Completion times (s) for individual sprints during the 8 × 25 m repeated sprint test.

	Sprint 1	Sprint 2	Sprint 3	Sprint 4	Sprint 5	Sprint 6	Sprint 7	Sprint 8
SB-ORAL	4.05 ± 0.26	4.10 ± 0.24	4.11 ± 0.21	4.14 ± 0.21	4.09 ± 0.17 *	4.12 ± 0.23 *	4.14 ± 0.22	4.12 ± 0.21 *
SB-LOTION	4.03 ± 0.26	4.07 ± 0.31	4.14 ± 0.29	4.11 ± 0.29	4.10 ± 0.25 *	4.12 ± 0.25 *	4.15 ± 0.28	4.13 ± 0.21 *
PLA	4.06 ± 0.26	4.13 ± 0.24	4.18 ± 0.24	4.20 ± 0.20	4.20 ± 0.21	4.23 ± 0.21	4.23 ± 0.21	4.23 ± 0.15

Values are presented as mean ± SD. SB-ORAL = oral sodium bicarbonate, SB-LOTION = topical sodium bicarbonate, PLA = placebo; * faster than PLA (*p* < 0.05).

**Figure 3.**
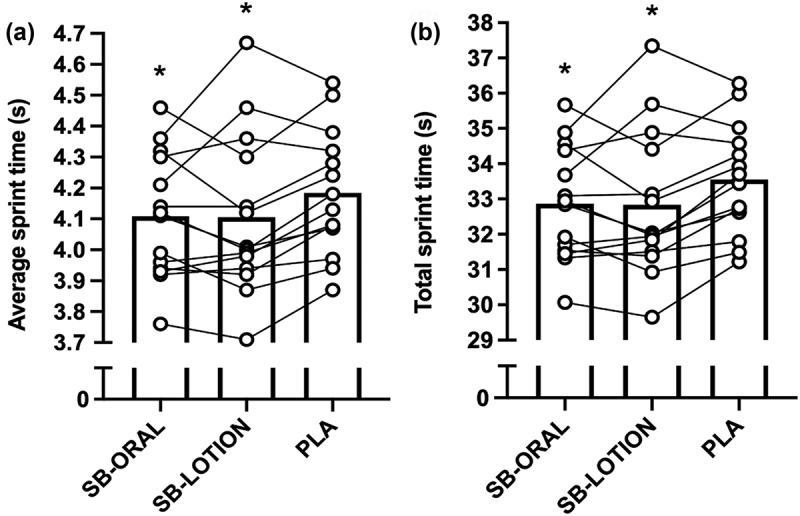
(a, b) 8 × 25 m repeated sprint test average times (a) and total times (b). Bars represent mean values. Individual treatment differences depicted by symbol/line. SB-ORAL = oral sodium bicarbonate, SB-LOTION = topical sodium bicarbonate, PLA = placebo; * faster than PLA (*p* < 0.05).

There were significant effects of treatment on average (*p* = 0.025, *η*_*p*_^*2*^ = 0.247) and total (*p* = 0.024, *η*_*p*_^*2*^ = 0.250) but not fastest (*p* = 0.227, *η*_*p*_^*2*^ = 0.180) sprint times or decrement score (*p* = 0.091, *η*_*p*_^*2*^ = 0.169). The average sprint time was 1.8% faster for SB-ORAL compared with PLA (−0.08 s; *95% CI*: −0.14, −0.01; *p* = 0.023) and 1.9% faster for SB-LOTION compared with PLA (−0.08 s; *95% CI*: −0.15, −0.01; *p* = 0.036). Cohen’s *d* revealed small effects for SB-ORAL (*d* = 0.38, *95% CI*: 0.11, 0.65) and SB-LOTION (*d* = 0.34, *95% CI*: 0.08, 0.60). The total sprint time was 1.8% faster for SB-ORAL compared with PLA (−0.61 s; 95*% CI*: −1.13, −0.09; *p* = 0.020) and 2.0% faster for SB-LOTION compared with PLA (−0.64 s; *95% CI*: −1.24, −0.04; *p* = 0.036). Cohen’s *d* revealed small effects for SB-ORAL (*d* = 0.38, *95% CI*: 0.11, 0.65) and SB-LOTION (*d* = 0.34, *95% CI*: 0.08, 0.60). The average and total sprint times were similar for SB-ORAL and SB-LOTION (both p > *0.05;* Figure 3a,b).

There were no significant treatment * time interactions for average (*p = *0.922,*η*_*p*_^*2*^ = 0.017) or maximum (*p* = 0.815, *η*_*p*_^*2*^ = 0.029) CMJ height. There were also no significant treatment effects for average (*p* = 0.607, *η*_*p*_^*2*^ = 0.038) or maximum (*p* = 0.746, *η*_*p*_^*2*^ = 0.022) CMJ height.

### 3.2. Blood acid–base balance and electrolytes

**Figure 4.**
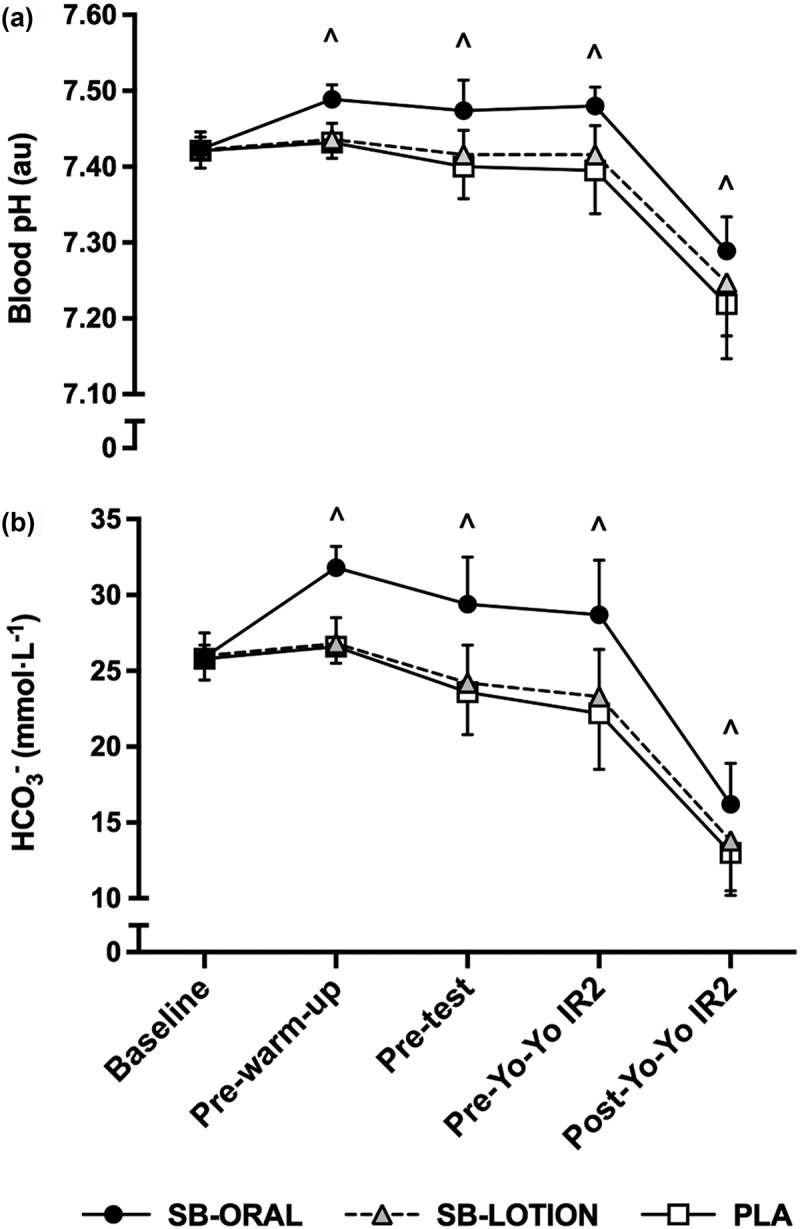
(a, b) Mean ± SD responses for blood acid–base balance (a, pH; b, bicarbonate). Bars represent mean values. Some SD error bars were removed for clarity. SB-ORAL = oral sodium bicarbonate, SB-LOTION = topical sodium bicarbonate, PLA = placebo; ^ elevated for SB-ORAL compared with SB-LOTION and PLA (*p* < 0.05).

There were significant treatment * time interactions for blood pH (*p* < 0.001, *η*_*p*_^*2*^ = 0.333) and HCO_3_^−^ (*p* < 0.001, *η*_*p*_^*2*^ = 0.516). Blood pH and HCO_3_^−^ were elevated for SB-ORAL compared with SB-LOTION pre-warm-up (+0.05 au., +5.1 mmol·L^−1^; both *p* < 0.001), pretest (+0.06 au., +5.3 mmol·L^−1^; *p* = 0.001, *p* < 0.001), pre-Yo-Yo IR2 (+0.06 au., +5.3 mmol·L^−1^; both *p* < 0.001) and post-Yo-Yo IR2 (+0.04 au., +2.4 mmol·L^−1^; *p* = 0.019, *p* = 0.007). Blood pH and HCO_3_^−^ were elevated for SB-ORAL compared with PLA pre-warm-up (+0.06 au., +5.3 mmol·L^−1^; both *p* < 0.001), pretest (+0.08 au., +5.8 mmol·L^−1^; both *p* < 0.001), pre-Yo-Yo IR2 (+0.09 au., +6.5 mmol·L^−1^; both *p* < 0.001) and post-Yo-Yo IR2 (+0.07 au., +3.2 mmol·L^−1^; *p* = 0.003, *p* < 0.001). No significant differences in blood pH and HCO_3_^−^ were observed for SB-LOTION compared with PLA pre-warm-up (+0.01 au., +0.2 mmol·L^−1^; *p* = 0.969, *p* = 1.000), pretest (+0.017 au., +0.6 mmol·L^−1^; *p* = 0.421, *p* = 1.000), pre-Yo-Yo IR2 (+0.021 au., +1.1 mmol·L^−1^; *p* = 0.527, *p* = 0.449) or post-Yo-Yo IR2 (+0.03 au., +0.9 mmol·L^−1^; *p* = 0.178, *p* = 0.745) (Figure 4a, b).

There was a significant treatment * time interaction for blood lactate (*p* = 0.001, *η*_*p*_^*2*^ = 0.280) but not Na^+^ (*p* = 0.244, *η*_*p*_^*2*^ = 0.092) or K^+^ (*p* = 0.166, *η*_*p*_^*2*^ = 0.103). There were significant treatment effects for Na^+^ (*p* < 0.001, *η*_*p*_^*2*^ = 0.466) and K^+^ (*p* = 0.030, *η*_*p*_^*2*^ = 0.237). Post Yo-Yo IR2 blood lactate was higher for SB-ORAL compared with SB-LOTION (+2.65 mmol·L^−1^; *p* = 0.009) and PLA (+2.42 mmol·L^−1^; *p* = 0.026) but was similar between SB-LOTION and PLA (−0.23 mmol·L^−1^; *p* = 1.000). Blood Na^+^ was elevated for SB-ORAL compared with SB-LOTION and PLA pre-warm up (+1 mmol·L^−1^, +2 mmol·L^−1^; *p* = 0.036, *p* < 0.001) and compared with PLA pretest (+2 mmol·L^−1^; *p* = 0.011), pre-Yo-Yo IR2 (+3 mmol·L^−1^; *p* = 0.008) and post-Yo-Yo IR2 (+2 mmol·L^−1^; *p* = 0.016). Pre-warm-up blood K^+^ was lower for SB-ORAL compared with SB-LOTION (−0.4 mmol·L^−1^; *p* = 0.019) and PLA (−0.4 mmol·L^−1^; *p* = 0.003). No significant differences in blood Na^+^ and K^+^ were observed for SB-LOTION compared with PLA pre-warm-up (+1 mmol·L^−1^, −0.1 mmol·L^−1^; *p* = 0.449, *p* = 1.000), pretest (+1 mmol·L^−1^, −0.04 mmol·L^−1^; *p* = 0.647, *p* = 1.000), pre-Yo-Yo IR2 (+1 mmol·L^−1^, −0.2 mmol·L^−1^; *p* = 0.804, *p* = 0.711) or post-Yo-Yo IR2 (+1 mmol·L^−1^, −0.3 mmol·L^−1^; *p* = 1.000, *p* = 0.458) (Table 2).

**Table 2.** Blood lactate and electrolyte response throughout the battery of team sport-specific exercise tests.

	Baseline	Pre-warm-up	Pre-test	Pre-sprints	Post-sprints	Pre-Yo-Yo IR2	Post-Yo-Yo IR2
**SB-ORAL**							
BLa^−^ (mmol·L^−1^)	1.16 ± 0.15	1.32 ± 0.33	2.63 ± 0.81	2.35 ± 0.46	7.13 ± 1.78	4.09 ± 1.93	13.87 ± 2.90 ^
Na^+^ (mmol·L^−1^)	135 ± 2	137 ± 2 *	137 ± 2 *	-	-	138 ± 3 *	139 ± 2 *
K^+^ (mmol·L^−1^)	4.4 ± 0.5	3.9 ± 0.3 *	4.0 ± 0.5	-	-	3.9 ± 0.4	3.8 ± 0.4
**SB-LOTION**							
BLa^−^ (mmol·L^−1^)	1.21 ± 0.28	1.36 ± 0.45	2.71 ± 1.06	2.41 ± 0.74	7.02 ± 2.43	3.82 ± 2.36	11.22 ± 2.48
Na^+^ (mmol·L^−1^)	135 ± 2	136 ± 2	136 ± 2	-	-	136 ± 3	137 ± 3
K^+^ (mmol·L^−1^)	4.3 ± 0.2	4.2 ± 0.4	4.2 ± 0.4	-	-	4.0 ± 0.4	3.9 ± 0.3
**PLA**							
BLa^−^ (mmol·L^−1^)	1.15 ± 0.28	1.31 ± 0.24	3.08 ± 1.68	2.55 ± 1.28	6.01 ± 2.45	4.20 ± 2.42	11.45 ± 2.55
Na^+^ (mmol·L^−1^)	134 ± 1	135 ± 2	135 ± 1	-	-	135 ± 2	136 ± 2
K^+^ (mmol·L^−1^)	4.3 ± 0.4	4.3 ± 0.3	4.2 ± 0.4	-	-	4.3 ± 0.7	4.2 ± 0.8

Values are presented as mean ±SD. Blood electrolytes were not measured pre- or post-sprints. SB-ORAL = oral sodium bicarbonate, SB-LOTION = topical sodium bicarbonate, PLA = placebo; * difference compared with PLA, ^ difference compared with SB-LOTION and PLA (*p* < 0.05).

### 3.3. Hydration status

**Figure 5.**
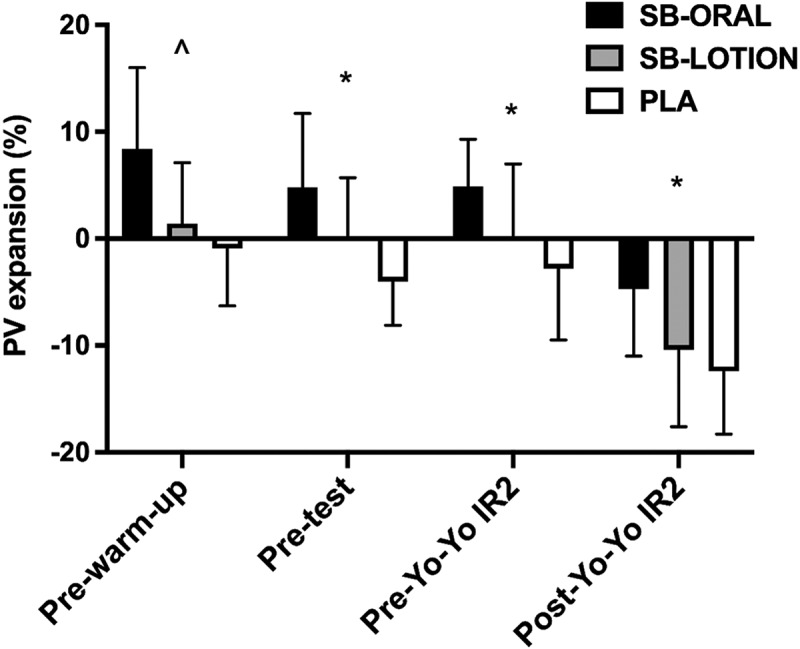
Mean ± SD for changes in plasma volume (PV) from baseline. Bars represent mean values. Some SD error bars were removed for clarity. SB-ORAL = oral sodium bicarbonate, SB-LOTION = topical sodium bicarbonate, PLA = placebo; ^ SB-ORAL higher than SB-LOTION and PLA, * SB-ORAL higher than PLA (*p* < 0.05).

There were significant effects of treatment on changes in plasma volume from baseline to pre-warm-up (*p* < 0.001, *η*_*p*_^*2*^ = 0.457), pretest (*p* = 0.002, *η*_*p*_^*2*^ = 0.392), pre-Yo-Yo IR2 (*p* = 0.003, *η*_*p*_^*2*^ = 0.363), and post-Yo-Yo IR2 (*p* = 0.003, *η*_*p*_^*2*^ = 0.356). Changes in plasma volume from baseline to pre-warm-up were greater for SB-ORAL compared with SB-LOTION (+7.0%; *p* = 0.030) and PLA (+9.3%; *p* = 0.004). Plasma volume expansion from baseline was also elevated for SB-ORAL compared with PLA pretest (+8.9%; *p* = 0.006) and pre-Yo-Yo (+7.6%; *p* = 0.015). The overall decline in plasma volume from baseline to post-Yo-Yo IR2 was attenuated for SB-ORAL compared with PLA (−7.7%; *p* = 0.006) but was similar for SB-LOTION compared with PLA (−2.4%; *p* = 0.765; Figure 5).

There was a significant treatment * time interaction for urine osmolality (*p* = 0.042, *η*_*p*_^*2*^ = 0.171). Post-exercise urine osmolality was higher for SB-ORAL compared with PLA (+224 mOsmols·kgH_2_O; *p* = 0.015) but was not statistically different for SB-ORAL compared with SB-LOTION (+148 mOsmols·kgH_2_O; *p* = 0.131) or SB-LOTION compared with PLA (500 ± 197 vs. 424 ± 226 mOsmols·kgH_2_O; *p* = 0.918). Urine color was similar between treatments at baseline (*χ*^*2*^(2) = 0.140, *p* = 0.933) and pre-exercise (*χ*^*2*^(2) = 4.512, *p =* 0.105) but there was a significant treatment effect post-exercise (*χ*^*2*^(2) = 7.538, *p* = 0.002). Urine color was significantly darker post-exercise for SB-ORAL compared with PLA (median: 4.0 vs. 3.0; *Z* = 0.857, *p* = 0.023) but was not significantly different for SB-LOTION (median: 3.5) compared with SB-ORAL (*p* = 0.156) and PLA (*p* = 0.395).

### 3.4. Rating of perceived exertion

There was no significant treatment * time interaction for RPE during the repeated sprint test (*p* = 0.098, *η*_*p*_^*2*^ = 0.142) but there was a significant treatment effect (*p* = 0.015, *η*_*p*_^*2*^ = 0.278). RPE was lower for SB-LOTION compared with PLA after the fifth (−0.9 au.; *p* = 0.036), sixth (−1.1 au.; *p* = 0.012), and eighth (−1.4 au.; *p* = 0.040) sprints and for SB-ORAL compared with PLA after the 6^th^ (−0.9 au.; *p* = 0.039) sprint. Post-Yo-Yo IR2 RPE was similar between treatments (all *p* > 0.05; Table 3).

**Table 3.** RPE (au.) after each of the 8 × 25 m repeated sprints and post-Yo-Yo IR2.

	Sprint 1	Sprint 2	Sprint 3	Sprint 4	Sprint 5	Sprint 6	Sprint 7	Sprint 8	Yo-Yo IR2
SB-ORAL	10.7 ± 3.0	11.3 ± 2.8	12.5 ± 2.8	13.4 ± 2.6	14.0 ± 2.4	14.9 ± 2.2 *	15.5 ± 2.1	16.6 ± 2.0	19.0 ± 1.1
SB-LOTION	10.5 ± 3.1	11.7 ± 2.8	12.4 ± 2.8	13.1 ± 2.7	13.9 ± 2.3 *	14.6 ± 2.0 *	15.7 ± 1.9	16.1 ± 2.2 *	19.2 ± 0.8
PLA	10.8 ± 3.2	11.6 ± 3.0	12.7 ± 2.6	14.1 ± 2.3	14.8 ± 2.0	15.8 ± 1.6	16.4 ± 1.5	17.5 ± 1.2	19.1 ± 0.9

Values are presented as mean ± SD. RPE values were measured using the 6–20 Borg scale. SB-ORAL = oral sodium bicarbonate, SB-LOTION = topical sodium bicarbonate, PLA = placebo; * lower than PLA (*p* < 0.05).

### 3.5. Gastrointestinal discomfort, blinding, and expectations

GI side effects were reported by eight participants (57%) for SB-ORAL, six participants (43%) for SB-LOTION and seven participants (50%) for PLA. Aggregate GI discomfort scores were not significantly different between treatments at baseline (*χ*^*2*^(2) = 0.095, *p* = 0.953), post-consumption (*χ*^*2*^(2) = 4.414, *p* = 0.110), pre-warm-up (*χ*^*2*^(2) = 3.161, *p* = 0.206) and post-exercise (*χ*^*2*^(2) = 3.935, *p* = 0.140). Blinding was protected for SB-ORAL and PLA as the highest number of participants able to correctly identify treatments at each time-point was less than 50% expected by chance alone (SB-ORAL: 34%; PLA: 14%). There were no significant differences in the number of correct and incorrect treatment assignment ratings for SB-ORAL and PLA (*p* > 0.05). SB-LOTION was identified by 50% participants at each time point, with the number of correct ratings significantly greater than for PLA pre-exercise (*χ*^*2*^(2) = 8.137, *p* = 0.017). Participants suggested “thicker texture” (5/7) and/or “a strong cooling effect on application” (6/7) as their reasons for being able to identify SB-LOTION. There was an effect of treatment on expectations (*χ*^*2*^(2) = 18.184, *p* < 0.001). Expectations were higher for SB-LOTION (median: 3.0) compared with SB-ORAL (median: 1.0; *Z* = 0.583, *p* = 0.008) and PLA (median: 1.0; *Z* = 0.488, *p* = 0.025) but similar for SB-ORAL compared with PLA (*p = 0.663*).

## 4. Discussion

The aim of this study was to examine the effects of oral and topical NaHCO_3_ during a battery of team sport-specific exercise tests. Topical NaHCO_3_ (0.9036 g·kg^−1^ BM PR Lotion) improved 8 × 25 m repeated sprint performance by ~2% but had no significant effect on blood acid–base balance or electrolytes. Improvements in Yo-Yo IR2 and repeated sprint performance for oral NaHCO_3_ were likely due to elevated extracellular buffering, increased glycolytic flux and sustained excitation-contraction coupling. We propose that the faster repeated sprint times for PR Lotion might be attributed to an interaction between NaHCO_3_ molecules and menthol that intensified the localized cooling sensation, in turn reducing participants’ perception of effort and allowing them to exert greater effort during exercise. Further research is required to replicate our findings for PR Lotion and elucidate the physiological mechanisms responsible for ergogenic effects.

Yo-Yo IR2 performance improved by 21% for SB-ORAL compared to PLA, which represented a moderate effect and is congruent with evidence from a meta-analysis [35]. In contrast, SB-LOTION showed a small but statistically insignificant effect (7% greater total distance) compared to PLA. This is comparable to findings from McKay et al. [26] that demonstrated small, non-significant effects of PR Lotion during 3 × 30 s Wingate cycling tests and suggests that oral NaHCO_3_ remains the most effective supplementation strategy for improving Yo-Yo IR2 performance. There were no improvements in CMJ performance for SB-ORAL or SB-LOTION, supporting some previous NaHCO_3_ studies [19] but not others [36]. Discrepancies between studies could relate to differences in athlete cohort; we recruited a variety of team sport athletes meaning that there was a large variability in CMJ performance that may have decreased the likelihood of observing any treatment effects. Interestingly, SB-ORAL and SB-LOTION improved 8 × 25 m repeated sprint performance (total and average times) by ~2%. These results add to equivocal previous findings for the effect of NaHCO_3_ on RSA in team sport athletes [17–19] and provide evidence for the performance enhancing effects of PR Lotion during this exercise task. It is possible that we found improvements in repeated sprint performance for oral NaHCO_3_ because of the absence of GI discomfort [20], as team sport athletes often experience severe side-effects [18]. They might be at greater risk of suffering GI discomfort than other athletes (i.e. cyclists) as their higher body mass requires larger absolute NaHCO_3_ doses to achieve ergogenic benefits. PR Lotion could offer an alternative strategy for improving RSA in team sport athletes, but the effect on Yo-Yo IR2 and CMJ performance remains unclear. Since this is the first study to show ergogenic effects of PR Lotion, it is important future work replicates our findings and investigates whether performance benefits exist for other exercise modalities.

Pre-exercise changes in blood buffering capacity for SB-ORAL typically achieved the minimum ergogenic threshold (i.e. HCO_3_^−^ >5.0 mmol·L^−1^ for 71% participants) [9] and blood acid–base balance was elevated compared to PLA throughout. Our improvements in repeated sprint and Yo-Yo IR2 performance for SB-ORAL were likely attributed to greater HCO_3_^−^ buffering, which increased H^+^ efflux from the muscle and protected against declining intramuscular pH [10]. This is supported by significantly higher blood lactate post-Yo-Yo IR2 for SB-ORAL, suggesting that NaHCO_3_ ingestion may have upregulated glycolytic flux by preventing the inhibition of glycolytic enzymes such as glycogen phosphorylase and phosphofructokinase-1 [5,37]. While blood lactate is only an indirect measure of glycolytic flux, our ~2.5 mmol·L^−1^ difference was comparable to studies reporting greater Yo-Yo IR2 performance for NaHCO_3_ [15,16]. Additionally, ergogenic effects of SB-ORAL could be explained by altered electrolyte concentration. SB-ORAL significantly increased blood Na^+^ and reduced blood K^+^ concentrations compared to PLA pre-warm-up, which agrees with previous findings [11,12]. In theory, these changes may lead to upregulation of Na^+^/K^+^-ATPase activity that would increase excitation-coupling contraction and sustain force generating capacity of muscles [8,12]. Since exercise-induced losses in intramuscular K^+^ are one of the causes of depressed muscle excitability [8], attenuating reductions in intracellular K^+^ might be crucial for the ergogenic effects for NaHCO_3_. Interestingly, blood K^+^ was not significantly lower post-Yo-Yo IR2 for SB-ORAL compared to PLA. Participants covered greater distance for SB-ORAL, therefore we cannot disregard that a significant effect would have been shown if total work had been matched between conditions. It is also important to note that our results only reveal changes occurring within extracellular compartments, and not whether NaHCO_3_ altered electrolyte concentrations within contracting muscles.

No significant effects of SB-LOTION were observed for blood acid–base balance or electrolytes. The small, non-significant effect of SB-LOTION on blood lactate after the repeated sprint test (+1.01 mmol·L^−1^) may have contributed to performance benefits, whereas post-Yo-Yo IR2 blood pH was slightly elevated (+0.03 au.) for SB-LOTION compared with PLA despite participants covering more distance. Although not practically feasible, it is possible that a greater dose of PR Lotion would have increased the magnitude of any effects. Overall, our results add to findings by McKay et al. [26] and suggest that topical application of 0.9036 g·kg^−1^ BM PR Lotion is not able to effectively deliver NaHCO_3_ molecules into systematic circulation. Whilst we were unable to trace the absorption of NaHCO_3_ molecules from PR Lotion across the skin, it is logical that their negative lipophilicity (partition coefficient, −0.82) somewhat restricts their ability to penetrate lipid bilayers in the stratum corneum [38]. Transdermal drug delivery is also only effective when small doses (mg per day) are required [25]. If PR Lotion does allow NaHCO_3_ molecules to penetrate the stratum corneum, given the large doses of NaHCO_3_ ingested orally (~20–30 g), it is unclear whether enough NaHCO_3_ from PR Lotion could reach the bloodstream to elicit ergogenic changes in HCO_3_^−^ [9]. As such, we suggest an alternative mechanism was responsible for faster repeated sprint times during SB-LOTION.

One explanation could be a localized cooling sensation from menthol (~0.5%) in PR Lotion [39]. Menthol is a cyclic terpene alcohol that may improve exercise performance by inducing a “cooling” effect to the skin via stimulation of the membrane-bound ion channel transient receptor potential melastatin 8 [39]. PR Lotion contains proportionately less menthol than other topical menthol formulations [40,41], but a similar absolute amount might have been given as we administered considerably more PR Lotion (~0.4 g menthol; assuming average dose for SB-LOTION was ~74 g) than researchers have used when investigating menthol gels (i.e. 2 mL of ~3.5% menthol Biofreeze® gel [41]). We propose that NaHCO_3_ molecules in PR Lotion interacted with menthol to form a protective layer over the skin that intensified menthols’ “cooling” sensation. Support for this can be drawn from the 43% participants who reported a “strong cooling” effect for SB-LOTION, whereas participants did not suggest a “cooling” sensation for SB-ORAL or PLA despite our placebo lotion also containing ~0.5% menthol. Furthermore, RPE was significantly lower for SB-LOTION compared with PLA during the repeated sprint test, which agrees with previous findings for topically applied menthol [40]. Reductions in RPE after NaHCO_3_ indicate ergogenic benefits might be explained by centrally mediated mechanisms [15,42]. Traditionally, this has been underpinned by the deleterious effects of H^+^ on the force generating capabilities of muscles [7], whereby declining pH causes localized pain [42,43]. Ingesting NaHCO_3_ likely leads to peripheral alterations (i.e. fewer H^+^ in the muscle) that modulate activation of group III and IV muscle afferents, in turn reducing negative feedback from muscles and sustaining drive to motor neurons [43,44]. We propose that the “cooling” effect from menthol in PR Lotion induced a similar centrally acting mechanism that attenuated localized muscle pain [45], meaning participants’ perception of muscle discomfort was lower than their actual degree of muscle fatigue [41], which allowed them to exert greater effort during the repeated sprint test. Future work should attempt to further elucidate potential physiological mechanisms responsible for ergogenic benefits of PR Lotion.

Another novel finding from this study was the positive effect of oral but not topical NaHCO_3_ on hydration status, with the changes in plasma volume for SB-ORAL compared with PLA (~8%) similar to previous results [13,46]. Post-exercise urine osmolality and color were also significantly different for SB-ORAL compared with PLA. Ingestion of water alone is relatively ineffective as a hyperhydration strategy, as most fluid is lost via urine [47]. This may explain why lower urine osmolality and lighter urine color were observed for PLA. Adding substances such as NaHCO_3_ with a high osmotic load enhances fluid retention by increasing plasma osmolality and volume [46,47]. Interestingly, differences between supplementation strategies and our participants’ high body masses meant that the amount of Na^+^ consumed during SB-ORAL (~6.5 g; proportion of Na^+^ in NaHCO_3_ was 27%, assuming 1 mol NaHCO_3_ is 84 g·mol^−1^) was greater than previous NaHCO_3_ studies [13,46] and more than the ~4 g given for traditional hyperhydration aids [47]. Despite the high amount of Na^+^ consumed, we believe that NaHCO_3_ can be safely incorporated into hyperhydration strategies of team sport athletes (i.e. American football, soccer) as they can lose ~4–6 g of Na^+^ per hour during training [48,49]. Our results also reinforce that NaHCO_3_ ingestion might be an effective strategy for improving fluid retention and exercise performance during challenging thermal conditions [50]. Additional research is needed to determine the reproducibility of changes in hydration status after NaHCO_3_ supplementation.

There are methodological limitations that need to be considered when interpreting our results that should be addressed in the future. First, plasma volume changes from our study were based on indirect measures (e.g. hemoglobin and hematocrit). This approach has been used for estimating changes in plasma volume during maximal exercise [13,31,46], but future research investigating the effect of NaHCO_3_ on plasma volume expansion should aim to use direct evaluation techniques [51]. Second, the intensified cooling sensation from menthol in PR Lotion contributed toward 50% participants identifying SB-LOTION, which may explain why participants reported significantly higher expectations of positive outcomes for SB-LOTION. Interestingly, however, 71% participants unable to identify SB-LOTION still improved their repeated sprint performance, and therefore it is difficult to conclude that the ergogenic effects of PR Lotion can be attributed solely to greater expectations. Further work is required to determine the most efficacious strategy for blinding PR Lotion during randomized placebo-controlled trials examining sports performance.

## 5. Conclusions

Topical NaHCO_3_ (0.9036 g·kg^−1^ BM PR Lotion) improved 8 × 25 m repeated sprint times by ~2% but had no significant effect on Yo-Yo IR2 or CMJ performance compared to a placebo. Oral NaHCO_3_ improved Yo-Yo IR2 and repeated sprint performance, which can likely be explained by elevated HCO_3_^−^ buffering, increased glycolytic flux and sustained excitation–contraction coupling. PR Lotion had no significant effect on blood acid–base balance, suggesting that it did not effectively transport NaHCO_3_ across the skin into systematic circulation. Therefore, we propose that ~2% improvements in repeated sprint times for PR Lotion can be attributed to an interaction between NaHCO_3_ molecules and menthol that intensified the cooling sensation, subsequently reducing participants’ perception of effort and allowing them to exert greater physical effort during the 8 × 25 m repeated sprint test. In conclusion, PR Lotion appears to improve RSA in team sport athletes, but further research is required to elucidate the physiological mechanisms responsible for performance benefits.

## Data Availability

The datasets used and/or analyzed during the present study are available from the corresponding author on reasonable request https://shura.shu.ac.uk/.

## Publication statement

This manuscript has not been published elsewhere and has not been submitted simultaneously for publication elsewhere.
